# The complete chloroplast genome of *Rosa cymosa* (Rosaceae), a traditional medicinal plant in South China

**DOI:** 10.1080/23802359.2020.1781563

**Published:** 2020-06-24

**Authors:** Mingyan Ding, Miao Liao, Peiqian Liu, Guoqing Tan, Yiqing Chen, Shi Shi

**Affiliations:** a Shunde Polytechnic, Foshan, China; b Guangdong Key Laboratory for Innovative Development and Utilization of Forest Plant Germplasm, South China Agricultural University, Guangzhou, China

**Keywords:** Chloroplast genome, next generation sequencing, *Rosa cymosa*, phylogenetic relationships analysis

## Abstract

*Rosa cymosa* is a traditional medicinal and ornamental plant in China. Here, we report the complete chloroplast genome of *R. cymosa*. The chloroplast genome is 156,607 bp in length with 37.48% GC content, containing a small single-copy (SSC) region (18,763 bp), a large single-copy (LSC) region (85,722 bp), and a pair of inverted repeats (IRs: 26,061 bp each). A total of 139 genes were predicted, including 92 protein-coding genes, eight ribosomal RNA genes, and 39 tRNA genes. Phylogenetic analysis based on chloroplast genomes of 16 plant species shows that *R. cymosa* is closest to *R. chiensis* ‘Old Bush’ and *R. lucidissima*. These complete chloroplast genomes can be subsequently used for researches of Rosaceae.

*Rosa cymosa* Tratt. is an evergreen climbing or scandent shrub belongs to Rosaceae and is wildly distributed in the south region of China (Gu and Robertson 2003). *Rosa cymosa* is an important species with potential exploitation and utilization values. The roots are used in the treatment of diarrhea, rheumatoid arthritis, descensus uteri, and hemorrhage as a traditional Chinese herbal remedy (Takashi et al. 1993). The flowers are beautiful and fragrant with strong climbing ability of the branches, which can be used for vertical greening of gardens, buildings, etc. (Zappi and Taylor 1997; Liu et al. 2011). In this study, we assembled the complete chloroplast genome of *R. cymosa* and explored the phylogenetic relationship with other species in Rosacea family.

Fresh and young leaves of *R. cymosa* were collected from Nan’ao Island, Guangdong Province, China (N23°26’02.07”, E116°58’03.84”), and the voucher specimen was deposited in the Herbarium of South China Agricultural University (CANT) under the accession number 440523-190718-018. The total genomic DNA was extracted from fresh leaves using a modified CTAB method (Doyle and Doyle 1987). A genomic library consisting of an insert size of 300 bp was constructed using TruSeq DNA Sample Prep Kit (Illumina, USA), and sequencing was carried out on an Illumina HiSeq Nova platform (Guangzhou Jierui Biotech). 6 Gb raw data of 150 bp paired-end reads were obtained and further assembled using GetOrganelle (Jin et al. 2018). The cp genome annotation was accomplished using Geseq (Tillich et al. 2017) and then manually checked by comparison against the complete cp genome of *R. rugosa* (Genbank accession number: NC_044094). The complete chloroplast genome of *R. cymosa* was submitted to GenBank with the accession number MT471268.

The complete chloroplast genome of *R. cymosa* (MT471268) is 156,607 bp in size, containing a small single-copy (SSC) region (18,763 bp) and a large single-copy (LSC) region (85,722 bp), separated by a pair of inverted repeats (IRs: 26,061 bp each). The overall GC content of the cp genome is 37.48%. There are 139 genes reported, including 92 protein-coding genes, eight ribosomal RNA genes, and 39 tRNA genes.

For phylogenetic analysis, a maximum-likelihood (ML) tree was performed based on the complete chloroplast genome sequences of 16 species from Rosoideae subfamily of Rosaceae family, with *Eriobotrya seguinii* as outgroup. All of the sequences were downloaded from NCBI GenBank. The ML analysis was constructed by RAxML software (Stamatakis 2014) with 1000 bootstrap replicates. The ML tree (Figure 1) showed that *R. cymosa* was the sister clade to *R. chiensis* ‘Old Bush’ and *R. lucidissima*, the species from *Fragaria* and *Rubus* were clustered respectively as sister groups to *Rosa*. This finding could serve as a valuable genomic resource for genetic researches on Rosaceae in the future.

**Figure 1.**
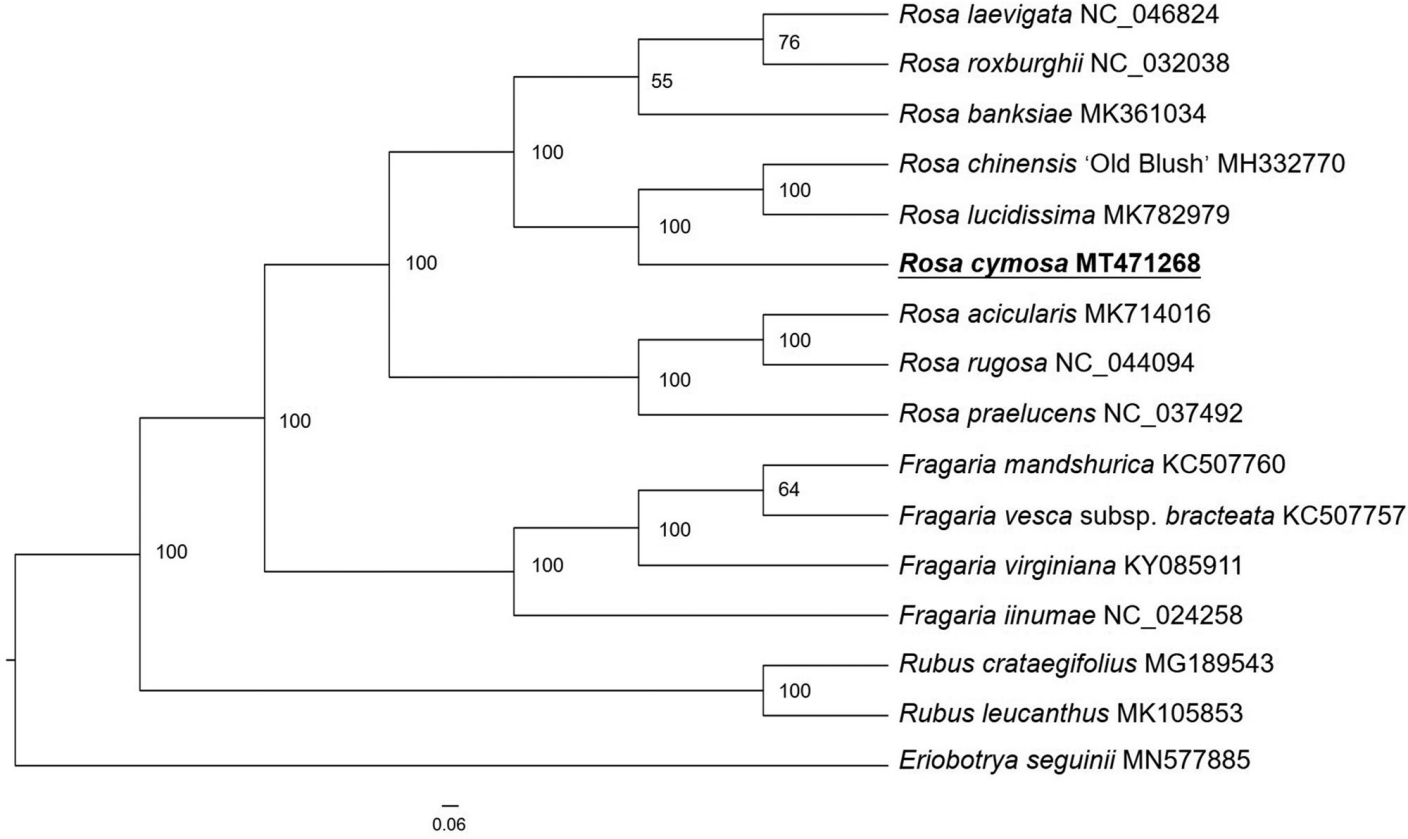
The maximum-likelihood (ML) analysis of 15 species of Rosoideae with *Eriobotrya seguinii* as outgroup based on chloroplast genome sequences. Numbers near the nodes are bootstrap support values.

## Data Availability

The data that support the findings of this study are openly available in NCBI at https://www.ncbi.nlm.nih.gov/, reference number MT471268.
